# Public fear of COVID-19 vaccines in Iraqi Kurdistan region: a cross-sectional study

**DOI:** 10.1186/s43045-021-00126-4

**Published:** 2021-07-27

**Authors:** Arazoo Issa Tahir, Dilkhosh Shamal Ramadhan, Ari Ahmed Taha, Rebar Yahya Abdullah, Saad Kazim Karim, Azad Karim Ahmed, Shalaw Faris Ahmed

**Affiliations:** 1 Bardarash Technical Institute, Nursing Department, Duhok Polytechnic University, Duhok Governorate, Duhok, Kurdistan Region Iraq; 2 College of Nursing, Nursing Department, Duhok University, Duhok Governorate, Duhok, Kurdistan Region Iraq; 3 Cardiac Center, Erbil Governorate, Erbil, Kurdistan Region Iraq; 4 Azadi Teaching Hospital, Duhok, Kurdistan Region Iraq; 5 Directorate of Health-Slemani Administration, Ministry of Health, Sulaymaniyah Governorate, Sulaymaniyah, Kurdistan Region Iraq; 6 Surgical Specialist Hospital-Cardiac Center Sulaymaniyah, Ministry of Health, Erbil Governorate, Erbil, Kurdistan Region Iraq

**Keywords:** Fear, COVID-19 vaccines, Kurdistan Region of Iraq

## Abstract

**Background:**

Vaccines remain one of the most effective methods to control infectious diseases; however, COVID-19 vaccines are challenging and novel. Vaccine support is still substantial in general, although vaccination fear has increased dramatically in recent decades. This is the first study aimed to determine the fear of the COVID-19 vaccination and the role of factors and reasons associated with fear in the Iraqi Kurdistan region.

**Results:**

A total of 1188 participants responded to the questionnaire about their fears of the COVID-19 vaccine. The majority of participants had a medium level of fear (56.7%). Fear was significantly (*p* < 0.001) associated with major demographic characteristics, social media use (51.8%), and losing family members, while other variables (previous seasonal influenza vaccine, previous infection, chronic medical diseases) show no relationship. Fear of side effects such as blood clotting was reported by the majority (45.03%) and indicated positive relation (*p* < 0.016). On the other hand, a high proportion, 39.9% and 34.01%, were afraid of AstraZeneca and Pfizer (*p* < 0.001), respectively; however, only about 4.63% had fear of Sinopharm.

**Conclusions:**

The fear of COVID-19 vaccination was widespread in the Iraqi Kurdistan region. In this way, fear was related to significant variables. To reduce the fear of vaccines and increase public acceptance, authorities and the Ministry of Health should initiate a public awareness campaign. As a result, the public health crisis will significantly improve.

## Background

The COVID-19 pandemic has already had a massive effect on communities around the world, with limits on travel and other preventive measures, including obligatory face coverings or quarantine being implemented to control the spreading of the virus [1, 2]. Nevertheless, it is known that such preventive steps may not be adequate to stop COVID-19 from spreading. As a result, developing and deploying the vaccine is among the most effective health intervention methods for preventing COVID-19 transmission [3–5]. Vaccination has been reported as one of the top notable public health achievements to have occurred during the twentieth century. It has resulted in the eradication of smallpox and control of poliomyelitis, measles, rubella, tetanus, diphtheria, and other infectious diseases [6]. The development of vaccines against COVID-19 has made rapid progress in the last year, and to date, three different vaccines showed good efficacy against COVID-19 [5, 7]. Positive results from clinical trials demonstrate that the COVID-19 vaccine is both safe and effective. The efficacy of a vaccine campaign, however, will be determined by population uptake rates. It is essential to begin planning and establishing successful vaccination strategies and marketing as soon as possible to ensure the highest possible uptake [8]. In the context of control of the COVID-19 pandemic, the willingness of the population in favor of vaccination may grow to hesitancy or fear from vaccination [9]. In general, vaccine support is still strong, although vaccination fear has grown significantly in recent decades. In some countries, this health anxiety has led to a rise in high refusal rates of vaccination. This has been linked to a fear of high threads of death from vaccine-preventable diseases [10]. In this context, the influence of media, particularly social media, appears to play a major role in the emergence of fear from the side effects of COVID-19 vaccines. Several studies have shown that fears remain a continuum of individual behavior and responses to every pandemic circumstance [5]. COVID-19 vaccines are now available in several countries, indeed, for Kurdistani people in Iraq. Early in March, the Health Ministry of the Kurdistan Region started an online registration system for the COVID-19 vaccine (https://vac.health.digital.gov.krd/?lang=en). Officially, vaccines manufacture by Pfizer, AstraZeneca, and Sinopharm are available in our locality. The general health workers and frontline health professionals as well as the elderly people will be given priority in the vaccination program. Therefore, in this exceptional circumstance, it is noteworthy that this is the first study aimed to determine the fear of the COVID-19 vaccination and the role of factors associated with fear in the Iraqi Kurdistan region.

## Methods

### Study design

In the Kurdistan Regional Government (KRG) of Iraq, a quantitative method was used to perform a descriptive cross-sectional online survey among Kurdish residents in four governorates (Erbil, Slemani, Duhok, and Halabja). All participants have been asked to agree to non-obligatory participation conditions via a well-formulated questionnaire preceded by consent on the Web-based Google platform. Participants had the option to withdraw from the study at any time. The planned research period was from April 6, 2021, to April 20, 2021. The questionnaire form was created by the researchers and uploaded via Google form and distributed online through platforms, namely, Emails, What’s up, Viber, and Facebook, over 2 weeks. A convenience sampling technique was applied to collect data from *n* = 1237 participants. However, only *n* = 1188 participants were included from the general population in our survey, and 49 responders were excluded due to incomplete replies to all questionnaire items. Ethical approval was obtained from the Scientific Committee at the College of Nursing, University of Duhok.

### Scales and assessments

The fundamental reasons behind this element of health anxiety disorder were to measure the degree of fear of vaccination against COVID-19 and to analyze the primary sources of information on the COVID-19 vaccines in the surveyed sample. For the identification of fear towards COVID-19 vaccination, we used multiple-choice format fear scales which were conceptualized and formulated by the researchers by a simple modification. Hence, based on an available review, 10-point Likert-type scales were chosen to assess the level of fear of vaccination. They had been successfully used in multiple cross-sectional studies analyzing vaccine fear, vaccine acceptance, and perceived risk of vaccination [2, 11–14]. On this scale, individuals are asked to provide a score between 0 and 10 for their level of fear of being vaccinated against SARS-Cov2, ranging from “0-no fear” to “10-very high level of fear.” After completion of data collection, the studied population was divided into three scores of fear which range from 0 to 3 “least fear”, 4 to 6 “medium fear”, and 7 to 10 “highest fear” (2). The questionnaire consists of sixteen short answer questions divided into three parts including independent variables such as age, gender, residential area, educational level, and occupation. In addition to the demographic data, some questions were asked to explore factors and reasons related to fear towards COVID-19 vaccination, as a second and a third part of the questionnaire were asked.

### Data analysis

The crossing between the three levels of fear intensity to identify variables associated with fear was calculated using a table of frequencies and percentages. The chi-square test was used to measure statistical significance for the entire work. Graphs were used to create the figures, and all *p* values (statistical significance *p* < 0.05) were determined. Version 23 of IBM SPSS Statistics was used (IBM Corporation, Armonk, New York, USA).

## Results

Table 1 shows that a total of 1188 participants were classified into three categories based on their fear of receiving the COVID-19 vaccine. Among them, 408 (34.3%) had a low level of fear, 674 (56.7%) had a medium level of fear, and 106 (8.9%) had a high level of fear. This implies that the majority of the participants were moderately afraid. When the tertiles were compared by variables (Table 1), we found that there were statistically significant variations by sex (*p* < 0.001). Women have a higher level of fear than men by 32.66% of medium level and 4.97% of high level. Participants aged 18–24, 25–34, and 35–44 were the most fearful than other ages (*p* < 0.002). Regarding the type of occupation, compared to other occupation categories, the majority of study participants were government employees (30.31%) and students (27.87%), and they had a highly significant association (*p* < 0.001) with dread. In terms of education, those with a diploma/bachelor’s degree had a higher level of fear, with a significant association (*p* < 0.002). The results demonstrated a statistically significant difference between provinces (*p* < 0.003): a high proportion of samples were from Duhok province 517 (43.51%), followed by Hawler 480 (17.08%).

**Table 1:** Levels of fear according to socio-demographic characteristics during the COVID-19 vaccination

Variables	Low, ***N*** (%)	Medium, ***N*** (%)	High, ***N*** (%)	Total	***p*** value
**General population**	408 (34.3%)	674 (56.7%)	106 (8.9%)	1188	
**Sex**					
Male	228 (19.19)	286 (24.07)	47 (3.96)	561	< 0.001**
Female	180 (15.15)	388 (32.66)	59 (4.97)	627	
**Age**					
18–24	120 (10.10)	243 (20.46)	47 (3.96)	410	< 0.002*
25–34	126 (10.60)	238 (20.03)	29 (2.44)	393	
35–44	116 (9.77)	128 (10.79)	17 (1.43)	261	
45–54	33 (2.78)	38 (3.19)	5 (0.42)	76	
55–64	11 (0.92)	25 (2.10)	7 (0.59)	43	
65+	2 (0.17)	2 (0.17)	1 (0.08)	5	
**Education**					
Lower than high school	24	33	10	67	< 0.002*
High school	33	35	11	79	
Diploma/bachelor	263	485	77	825	
H.diploma/Master/Ph.D	88	121	88	217	
**Occupation**					
Healthcare workers	117 (9.59)	148 (11.27)	14 (0.92)	279	< 0.001**
Government employee	128 (10.77)	203 (17.08)	27 (2.27)	358	
Self-employee	22 (1.85)	23 (1.93)	5 (0.42)	50	
Private sector	17 (1.68)	26 (3.36)	3 (0.42)	46	
Students	86 (10.21)	205 (17.25)	40 (3.36)	331	
Jobless	38 (3.19)	68 (5.72)	18 (1.51)	124	
**Province**					
Hawler	183 (15.40)	259 (21.80)	38 (3.19)	480	0.003*
Sulaymaniyah	50 (4.20)	103 (8.67)	6 (0.50)	159	
Duhok	168 (14.14)	288 (24.24)	61 (5.13)	517	
Halabja	7 (0.58)	24 (2.02)	1 (0.08)	32	

*χ*^*2*^ chi-square test; *p < 0.05 is significant; **p < 0.001 is highly significant

Table 2 indicates that the majority of the participants (60.35%) were not infected with COVID-19. Approximately 78% and 85% of the sample did not have a previous seasonal influenza vaccine or chronic diseases, respectively. Even though the majority of study participants (63.46%) did not lose a family member as a result of the COVID-19 pandemic, we discovered a statistically significant connection between fear and losing a family member. Meanwhile, more than half of the participants (51.85%) declared that the primary sources of information were social media/Internet, and we found a strong connection between sources of information and levels of fear (*p* > 0.001).

**Table 2:** Relevant responses to the COVID-19 vaccine

Variable	Number (***N***)	Percent (%)	***p*** value
Q6/ Have you been infected and diagnosed with COVID-19?			
Yes	471	39.64	0.204
No	717	60.35	
Q7/ Have you lost any family members due to COVID-19?			
Yes	434	36.53	0.001**
No	754	63.46	
Q8/ Do you suffer from a chronic medical condition?			
Yes	167	14.05	0.449
No	1020	85.85	
Q9/ Did you receive any previous seasonal influenza vaccine?			
Yes	263	22.13	0.216
No	925	77.87	
**Sources**			
Social media/Internet	616	51.85	0.001**
International organizations such as WHO/CDC	192	16.16	
Television	127	10.69	
Healthcare professionals	109	9.17	
Family/friends	67	5.63	
Government agencies	78	6.50	
Total	1188	100.00	

*χ*^*2*^ chi-square test; *p < 0.05 is significant; **p < 0.001 is highly significant

**Fig. 1:**
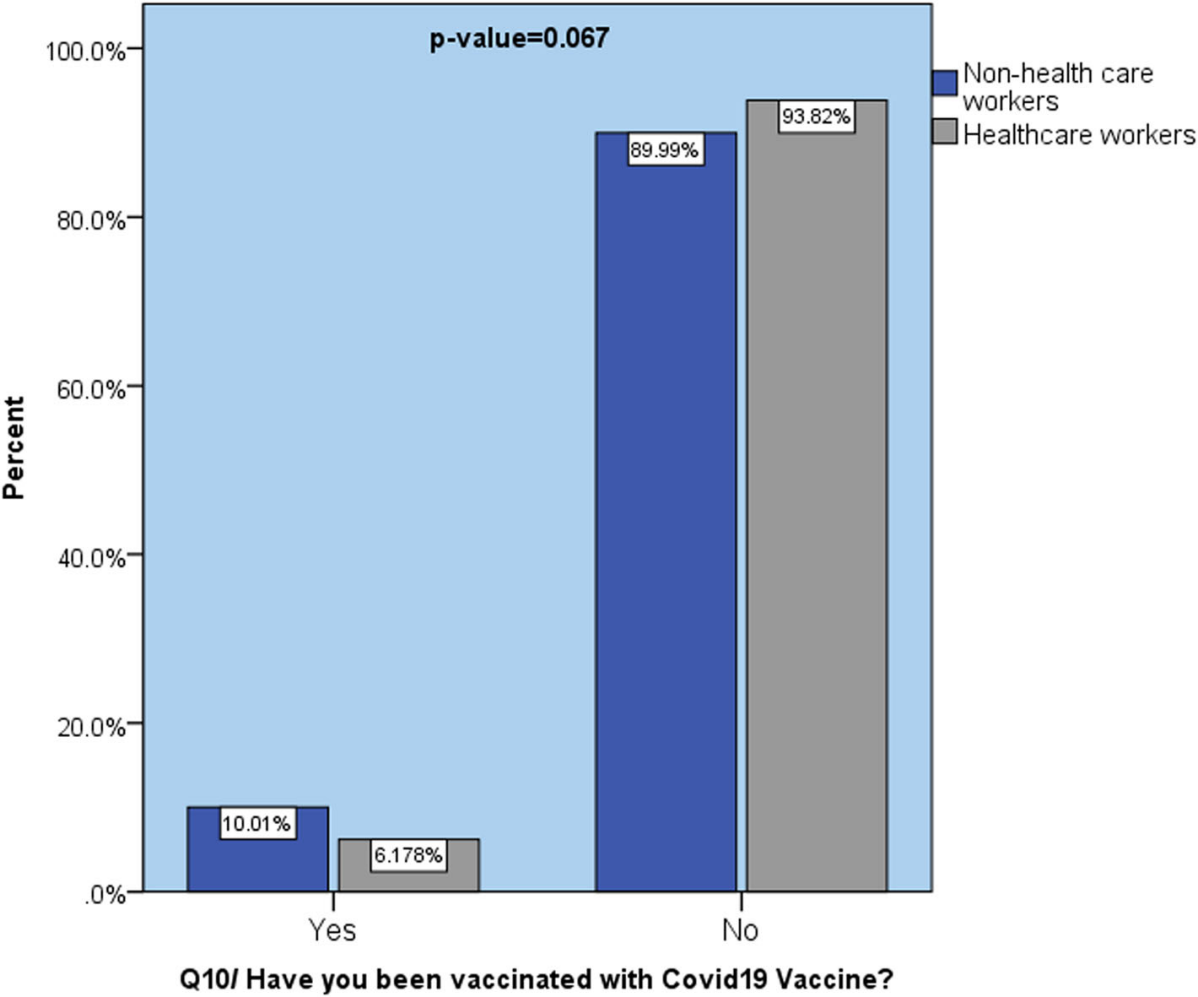
Comparison of COVID-19 vaccination among healthcare and non-healthcare workers

When comparing participants who were vaccinated with COVID-19 to those who were not, the vast majority of study respondents, including medical professionals (91.4%) and non-healthcare workers (90.65%), were not vaccinated during the period of this survey. However, healthcare workers were more willing and got vaccinated; a positive relation (*p* > 0.001) is identified in Fig. 1.

**Fig. 2:**
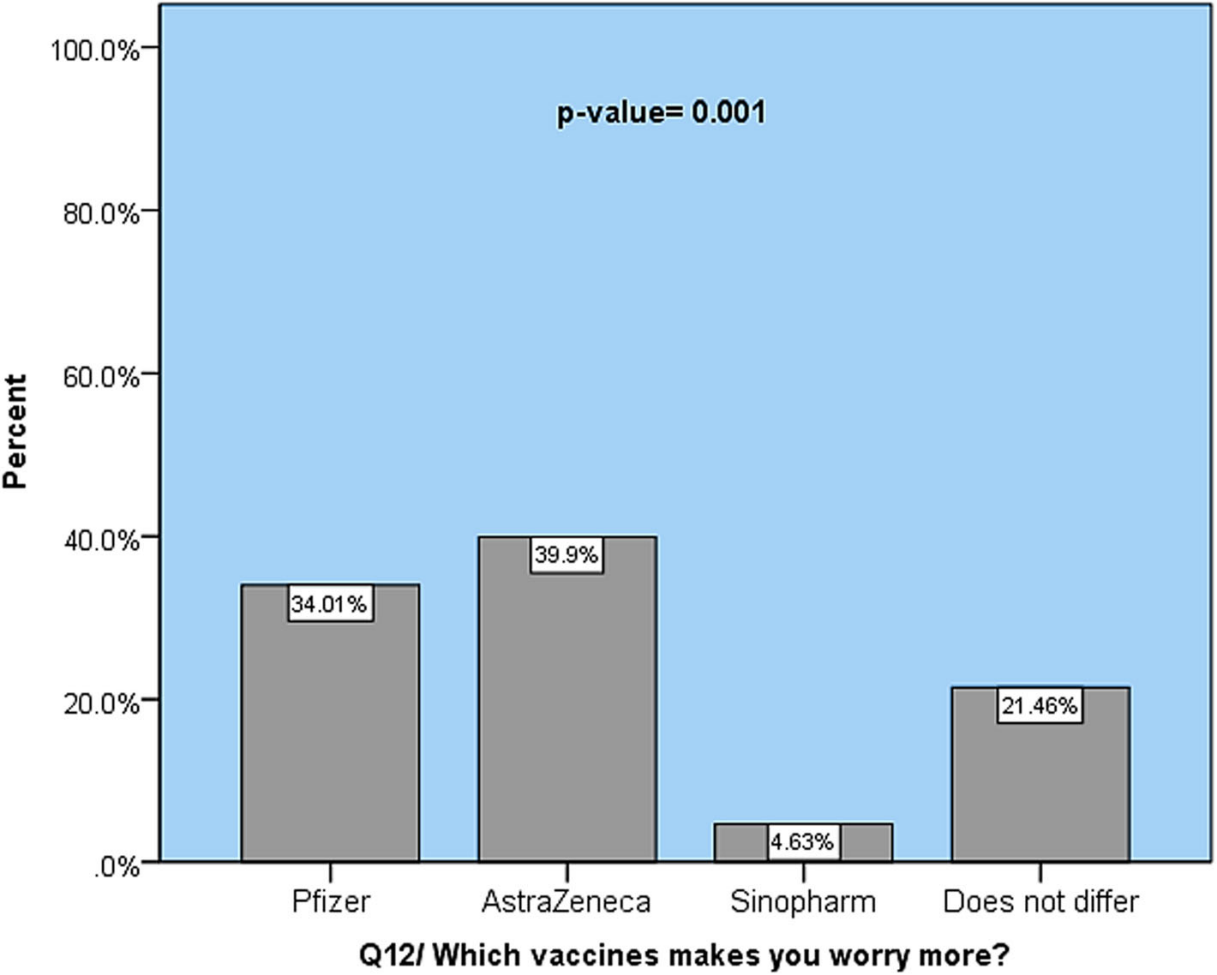
Public response to types of COVID-19 vaccines concerning fear

Concerning the association between fear and the type of COVID-19 vaccine, Fig. 2 shows that there is a significant relationship between types of vaccine and fear (*p* > 0.001). The AstraZeneca and Pfizer vaccines frightened the most people (39.9% and 34.01%, respectively).

**Fig. 3:**
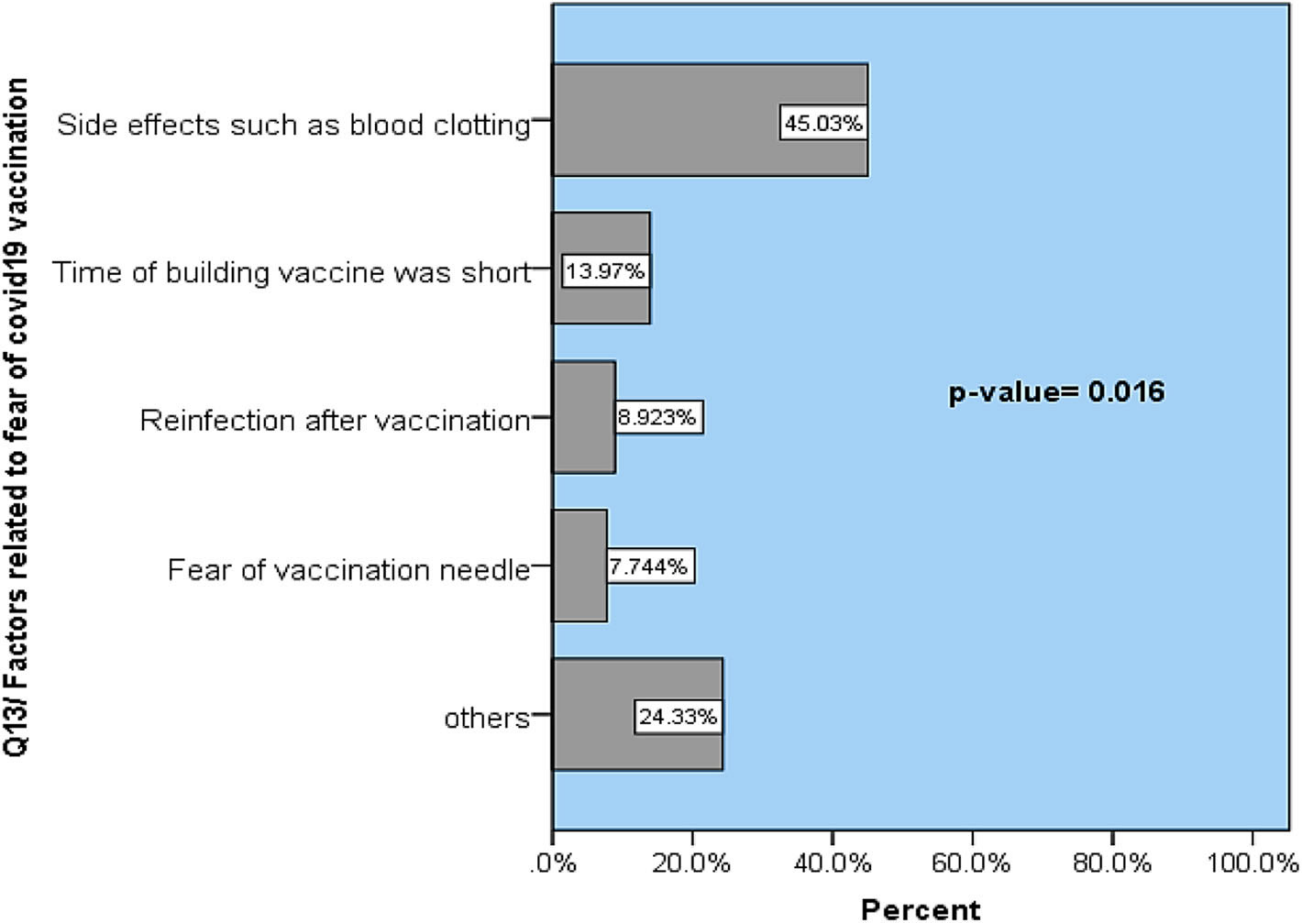
Reasons affecting fear of COVID-19 vaccination

In comparison to the other factors, 45.03% of samples reported fear of COVID-19 vaccines due to side effects, particularly blood clotting (*p* > 0.016) (Fig. 3.

## Discussion

On March 11, 2020, the World Health Organization declared the outbreak of COVID-19 as a pandemic disease [15]. Fear, anxiety, and hesitancy are all present in the current COVID-19 pandemic, which is thought to be an ideal environment for the spread of uncertainty among the population [16–19]. Vaccines have emerged as savior methods in the face of the world’s greatest health and economic crisis recently [20]. The present study aimed to determine fear of COVID-19 vaccination and their association with related factors. The results illustrate that women; people aged 18–24, 25–34, and 55–64; government employees; and students all had a high level of fear of the COVID-19 vaccine. Male and healthcare workers, on the other hand, were less afraid. Women experience more health anxiety than males as a result of a public health issue and they experience more fear, and this can be observed due to gender differences [21, 22]. Furthermore, women were the most affected in a study conducted in Peru among 3887 persons on the fear perception of the COVID-19 vaccination [2], as it was in Iraq [23], which is consistent with the ongoing study. Another notable finding in this study is that young people and mature adults were more fearful of the COVID-19 vaccines, which is consistent with other studies conducted during the COVID-19 pandemic in the Kurdistan area [24], Iraq [23], Austria [25], and the UK [26]. These age groups are using technology more than the elderly. This indicates that the younger generation is more vulnerable to depression, anxiety, and fear.

In our work, different factors were significantly (*p* value = 0.016) associated with fear of COVID-19 vaccination and the main reasons were fear of side effects such as coagulopathy and time of building the vaccines, while fear of needle was the least factor. This is in line with accumulated evidence that the major reason for hesitation or refusal of COVID-19 vaccines was fear of side effects [20, 27–29]. Many articles have been published in the scientific literature on the contents and side effects of vaccines [27, 30].

Importantly, we revealed that there was a strong relationship (*p* value = 0.001) between losing a family member and fear of COVID-19 vaccines, while previous COVID-19 infection, chronic illness, and obtaining seasonal influenza vaccine were not. Many studies during the COVID-19 pandemic observed a connection between fear of transmitting the disease and losing family members [31–33]. However, recent researches show the association between vaccine acceptance and other related variables such as previous influenza vaccination and having a history of chronic diseases, respectively [28, 34].

Social media is one of the main channels for updating COVID-19 information [35, 36]. Participants are frequently exposed to social media. Recent studies in the Kurdistan region have already shown the effect of social media on mental health during COVID-19 [24], and this study also discovered a major connection (*p* < 0.001) between fear of vaccine use and social media. To address this phenomenon, policymakers, regulators, the Ministry of Health, education, and media professionals should cooperate, and only data that has been thoroughly reviewed should be made accessible to the general population. Furthermore, fear was strongly (*p* = 0.001) associated with types of available vaccines, with the vast majority have fear of AstraZeneca, Pfizer, and Moderna, respectively. In a study of *n* = 1020 participants conducted in Poland, Pfizer and Moderna received a high level of trust, while Oxford/AstraZeneca received a low level of trust [14]. There are some possible explanations for the AstraZeneca vaccine’s apprehension. Firstly, knowledge regarding AstraZeneca’s side effects was more widely disseminated than other forms [37, 38]; second, the media and expert groups paid more attention to the mechanism of action of mRNA vaccines, resulting in a higher degree of understanding and acceptance [14]. We also observed that only about 9.2% of participants were vaccinated at the time of the survey, with healthcare professionals being the most enthusiastic about the vaccine program and engaging in it [29, 39, 40]. High intention to obtain COVID-19 vaccines if they were available has been reported by [1, 41, 42], which disagrees with the results of the ongoing research.

### Limitations

This research has some limitations that should be highlighted. To begin with, since our study is a cross-sectional one, we can only show you a snapshot of vaccine anxiety at one point in time. Second, due to the restrictions and measurements taken during the COVID-19 pandemic, this is an online questionnaire survey that may affect the generalizability of the sample. Thus, since it is an Internet-based survey, the majority of respondents were young adults, and the elderly had fewer opportunities to participate. Third, since this study was conducted at the beginning of the COVID-19 vaccination campaign when people were fearful of its use because it was new, they may respond differently when the vaccination campaign became part of the policymakers, Ministry of Health, and educational strategies. Furthermore, there was a chance of selection bias due to online participation in which a certain group of people can participate rather than different categories in the community. The study was also prone to external validity as the population we chose is those who use the Internet while those who do not use the Internet did not have a chance to be included in the current study.

## Conclusions

In the present study, we conclude that fear of COVID-19 vaccines is widespread among Kurdish people. Female sex, younger ages, losing family members, social media use, vaccine side effects, and types of vaccine are strongly associated with fear, while male sex, healthcare workers, and other variables are not associated with fear. According to our results, more psychological and physical preparedness is required to deal with health emergencies, and authorities and the Ministry of Health should address and develop these mental issues.

## Data Availability

The datasets that were generated during and/or analyzed during the current study are available from the corresponding author on reasonable request.

## Abbreviations

COVID-19: Coronavirus disease 2019
SARS: Severe acute respiratory syndrome coronavirus 2
mRNA: Messenger ribonucleic acid
KRG: Kurdistan Regional Government
